# Moving beyond disclosure: rethinking universal support for neurodivergent employees

**DOI:** 10.3389/fpsyg.2025.1547877

**Published:** 2025-05-22

**Authors:** Hanna Kalmanovich-Cohen, Steven J. Stanton

**Affiliations:** Department of Management and Marketing, Oakland University, Rochester, MI, United States

**Keywords:** neurodiversity, workplace inclusion, disclosure, universal design, inclusive language, employee lifecycle

## Introduction

Neurodivergent individuals represent an estimated 15-20% of the global population (Doyle, 2020), yet they continue to face significant barriers to workplace inclusion. Approximately 85–90% of neurodivergent individuals experience unemployment or underemployment (Krzeminska et al., 2019). While early disclosure of neurodivergence can improve equity in recruitment, selection, and retention, many candidates hesitate to disclose their neurotype due to concerns about stigma and negative reactions (Doyle, 2020). For neurodivergent individuals to become better integrated into the workforce, solutions are necessary, and one potential strategy is to facilitate and encourage disclosure. Recent research demonstrates that even minor changes—such as modifying the language on a disclosure form—can increase the likelihood of disclosure of a disability (Santuzzi et al., 2024). However, disclosure alone may not be enough to address the structural and cultural challenges neurodivergent individuals face at work. This raises two key questions: First, how and when should organizations facilitate disclosure of neurodivergence? Second, what can organizations do, beyond facilitating disclosure, to create inclusive and supportive environments for neurodivergent employees?

To address these questions and offer actionable insights, we start by examining how different disability frameworks—such as the medical, social, biopsychosocial, and strength-based models—shape organizational language, practice, and culture surrounding disclosure. Each framework promotes distinct language to describe neurodivergent individuals, and the language used can directly influence whether individuals choose to disclose (Santuzzi et al., 2024).

From this, we argue that while disclosure can provide access to tailored accommodations, disclosure should not be the sole pathway to support neurodivergent employees. Not all neurodivergent individuals will disclose and relying exclusively on disclosure risks alienating those individuals. Many inclusive practices, such as flexible scheduling or quiet workplaces, could be made universally available, benefiting all employees regardless of disclosure. At the same time, we recognize that universal support may not meet every individual's specific needs. Personalized accommodations will still be necessary in some cases, and disclosure will remain important in addressing these accommodations. Therefore, we advocate a balanced approach—one that reduces reliance on disclosure by implementing universal support, while also fostering a culture that makes voluntary disclosure safe.

We examine how disclosure and universal support operate across the employee lifecycle, from recruitment to retention. We emphasize that language and organizational practices must evolve in tandem to support neurodivergent individuals at every stage of their careers. However, without a genuine and organization-wide shift in culture, even well-intended policy changes may prove ineffective.

## Neurodivergence disclosure language

Despite growing interest in neurodiversity in organizational research, the language used to describe neurodivergent individuals remains inconsistent. Terms such as “impairment,” “disability,” and “condition” are often used interchangeably, yet each carries distinct connotations. This lack of consistency reflects underlying conceptual frameworks used to conceptualize neurodiversity and can influence whether individuals feel safe disclosing their neurodivergent identity (Santuzzi et al., 2024).

The medical model of disability views neurodivergence as a disorder or deficit, often implying a need for treatment or accommodation for the individual (LeFevre-Levy et al., 2023). Language associated with this model, such as “disorder,” “deficit,” or “impairment,” frames neurodivergence as a deviation from a normative standard. However, such terms may not align with how neurodivergent individuals perceive their own identities and can discourage disclosure (Buijsman et al., 2023). A practical step beyond the medical model would be to modify the narrow language on the Voluntary Self-Identification of a Disability (Office of Federal Contract Compliance Programs., 2017) form, commonly used by United States organizations. Expanding the list of qualifying neurotypes to include not only clinically diagnosed conditions (e.g., major depression, bipolar disorder) but also non-pathological neurotypes (e.g., dyslexia, sensory processing differences) would reflect a shift toward a more inclusive understanding of disability.

In contrast, the social model of disability refers to neurodivergent individuals as having a disability (Dwyer, 2022). But this disability is not viewed as a personal impairment, rather it stems from the organization's failure to accommodate the individual's unique needs (LeFevre-Levy et al., 2023). This model encourages terms like “disability” but emphasizes environmental changes and a need for support, such as modifying job duties or offering flexible schedules, to foster inclusion rather than stigmatization (LeFevre-Levy et al., 2023). However, simply changing language is not enough to remove the deep-rooted stigma or negative beliefs that may still be present in an organization's culture, which can discourage disclosure (Hassard et al., 2024). Therefore, changes in terminology must be accompanied by policy and practice shifts. One recommendation is to incorporate more inclusive language into recruitment materials, job descriptions, and performance reviews. For example, using terms like “neurotype” instead of “disability” can empower candidates and employees to disclose.

More recent models of neurodiversity emphasize the unique strengths neurodivergent individuals bring to the workplace (Doyle, 2024). The biopsychosocial model (Doyle, 2020), integrates biological, social, and psychological factors, encouraging organizations to create an environment that supports both employee wellbeing and organizational success. Similarly, the strength-based neurodiversity model (Fung, 2024) reframes neurodiversity as a natural and valuable form of human diversity, emphasizing the unique strengths of some neurodivergent individuals, such as creativity, attention to detail and pattern recognition (Doyle, 2024). These frameworks tend to use terms like “condition” to describe neurodivergent individuals, reflecting a shift away from the stigmatized focus on “disability” (Doyle, 2024).

However, from a neurodiversity-affirming perspective, even the term “condition” can imply deviation from a norm (Chapman, 2021). Alternatively, referring to neurodivergent traits as “neurotypes” might better reflect the view of cognitive differences as natural human variations (Doyle, 2024). This linguistic shift has practical implications for workplace inclusion. Using less stigmatized language can facilitate disclosure (Santuzzi et al., 2024), by presenting neurodivergent traits more positively. A key recommendation is for organizations to reframe neurodivergence not as something requiring accommodation, but as a normal form of cognitive diversity that enriches the workplace (Fung, 2024).

However, language alone is not enough. While the way organizations talk about neurodiversity can influence disclosure, it is their everyday practices and culture that ultimately determine whether individuals feel safe to share—and whether they receive the support they need as a result (Saleh et al., 2023). For example, the United Kingdom's “Disability Confident” scheme offers interview guarantees to candidates who disclose a disability. While widely adopted, “Disability Confident” has not significantly improved disability representation or outcomes (Hoque et al., 2024). This suggests that surface-level initiatives, even when framed in inclusive language, are insufficient without accompanying changes to organizational policies and culture.

## Balancing disclosure and universal support

While inclusive language can signal organizational commitment to neurodiversity, it is not sufficient on its own. To create truly inclusive environments, organizations should proactively design workplaces that are flexible, accessible and supportive for everyone. Rather than treating support as something triggered by disclosure, organizations can adopt universal design principles that make workplaces accessible by default (Doussard et al., 2024). These practices, such as offering flexible work hours, quiet spaces, job-sharing options, and noise-canceling headphones, accommodate a wide range of work preferences and cognitive needs without requiring individuals to disclose their neurotype (Doyle, 2020; Sargent, 2025). Additionally, tools like note taking software or the ability to record meetings can support employees with learning differences (Sargent, 2025). When offered proactively, these accommodations not only benefit neurodivergent employees but also enhance the overall work experience for everyone.

This raises an important question: can some benefits of disclosure, such as tailored support, be achieved without requiring disclosure at all? While disclosure remains necessary for certain individualized accommodations, many common needs can be met through universal support. By framing disclosure as voluntary, organizations reduce pressure on individuals to disclose and demonstrate their commitment to supporting all employees, regardless of their decision to disclose. This approach encourages disclosure when more personalized assistance is needed beyond what universal support provides.

The decision to disclose is often influenced by the visibility or invisibility of neurodivergence (Santuzzi et al., 2024). When neurodivergence is visible, others may assume they understand the individual's needs and see disclosure as unnecessary, but these assumptions are often oversimplified or inaccurate. In contrast, when neurodivergence is invisible or concealable, individuals may be reluctant to disclose unless they perceive the environment as genuinely supportive. If disclosure remains the only path to support, organizations risk overlooking or misjudging neurodivergent talent (Doyle, 2020). Universal design helps mitigate these risks by addressing a broad range of needs without requiring individuals to disclose. To be effective, however, universal support must be embedded throughout the entire employee experience, from recruitment to retention. The next section examines how disclosure and universal support can work together across the employee lifecycle.

## Disclosure and universal support across the employee lifecycle

Disclosure remains important throughout the employee lifecycle, influencing when and how organizations can offer tailored support. At each stage, from recruitment to retention, the language that organizations use influences whether candidates and employees feel comfortable disclosing their neurodivergent identity (Santuzzi et al., 2024). While changing language on disclosure forms is a helpful starting point, inclusive practices should be embedded throughout the employee experience, regardless of whether disclosure occurs. This includes both tailored accommodations made possible through disclosure and universal support that benefits all employees (see Table 1 for summary).

**Table 1:** Summary of recommendations for supporting neurodivergent employees across the employee lifecycle.

**Stage of employee lifecycle**	**Strategy type**	**Key recommendations**	**Potential impact**
Recruitment and selection	Disclosure	Use inclusive language in job materials (e.g., “minority neurotypes”) and emphasize that disclosure is voluntary and used to enable support	Signals commitment to inclusion, attracts a wider pool of candidates and encourages voluntary disclosure
	Disclosure	Explain how disclosure can facilitate personalized accommodations during recruitment and assessment	Builds trust and reduces stigma around neurodiversity
	Universal support	Offer varied interview formats and clearly communicate assessment expectations to all candidates	Promotes fairness by supporting different cognitive styles and reducing reliance on disclosure
	Universal support	Provide accessible alternatives to traditional assessments (e.g., written, oral, or task-based formats)	Ensures all candidates can demonstrate their strengths effectively
Training and development	Disclosure	Integrate neurodiversity awareness training for managers and teams	Fosters a culture of understanding and inclusion, increasing psychological safety
	Disclosure	Offer mentorship and coaching tailored to neurodivergent needs	Supports development and belonging for employees who disclose
	Universal support	Apply universal design to training (e.g., visual/audio/written materials, self-paced modules)	Enhances learning outcomes and accessibility for all employees
Retention	Disclosure	Regularly gather feedback on inclusion and accommodation policies and adapt them over time	Ensures support remains responsive and relevant to employee needs
	Universal support	Normalize flexible work practices (e.g., quiet spaces, flexible schedules, asynchronous communication)	Promotes well-being and performance without requiring disclosure
	Universal support	Create flexible, strength-based career development opportunities	Increases retention by enabling employees to grow in ways that align with their strengths

### Recruitment and selection

During planning and recruitment, organizations can benefit from attracting neurodivergent candidates by proactively communicating their commitment to inclusion. One actionable recommendation is to use inclusive language in job descriptions (e.g., minority neurotypes), which signals support for neurodivergent applicants and explains how disclosure can lead to personalized accommodation (Saleh et al., 2022). This is particularly important as securing a job is a common challenge for neurodivergent individuals (Krzeminska et al., 2019).

In the selection process, disclosure may allow for flexible assessment tools, such as virtual interviews or alternative assessment formats (e.g., written, oral, or practical tests). These accommodations allow neurodivergent candidates to better demonstrate their abilities, ultimately increasing their chances of being hired (Chang et al., 2023). However, organizations should also implement universal practices, such as offering interview format choices or clearly describing assessment expectations, even without disclosure. This promotes equity in hiring by supporting a broad range of cognitive styles and reduces the need for candidates to self-identify.

### Training and development

Disclosure can support the design of personalized training programs that accommodate diverse learning styles (Campanaro et al., 2021). An actionable recommendation is to incorporate neurodivergent-inclusive language into ongoing training for managers and colleagues (Lauder, 2024). This ensures awareness across the organization and helps create an environment where all employees feel supported. By fostering a shared understanding of neurodivergence, organizations not only reduce stigma but also strengthen workplace culture. It promotes an environment that values neurodiversity and supports the development of personalized career paths aligned with employees' strengths. Additionally, organization-level awareness can encourage mentoring opportunities, connecting neurodivergent employees with mentors who understand their unique needs or share similar experiences (Ezerins et al., 2024).

Simultaneously, universal design principles should guide all training programs. For example, offering all training materials in multiple formats (e.g., visual, audio, and written), allowing self-paced modules, or minimizing sensory overload in training environments ensures broader accessibility (Izzo et al., 2008). These universal features improve learning outcomes for all employees, not just those who disclose a need.

### Retention

Long-term inclusion requires more than initial accommodations—it demands sustained cultural and structural support. Organizations that normalize neurodiversity through inclusive development programs and flexible career pathways can increase employee satisfaction and retention (Kersten et al., 2025). An actionable recommendation is to regularly solicit feedback on policies and accommodations (Waisman-Nitzan et al., 2021), ensuring that support systems evolve as employee needs change.

In addition, universal retention strategies, like allowing flexible schedules, providing quiet workspaces, or implementing asynchronous communication norms (Kalmanovich-Cohen and Stanton, 2024), can improve wellbeing for all employees while removing the need for disclosure. These practices foster a work environment where neurodivergent individuals can succeed without needing to justify or explain their differences.

## Conclusion

The challenge for organizations is to strike a balance between encouraging disclosure to provide personalized accommodations and offering universal support that benefits all employees, regardless of disclosure. Neurodiversity, by its very nature, encompasses a wide range of neurotypes, each with unique needs (Doyle, 2020). While disclosure can help ensure that support is customized, it should not be the sole path to inclusion. Creating a truly inclusive workplace means embedding both language and practices that affirm neurodivergent experiences throughout the employee lifecycle. This includes adopting less stigmatizing terminology, fostering psychologically safe environments for disclosure, and designing universally accessible workspaces and processes. Organizations can proactively implement universal accommodation, creating an environment which supports all employees while reducing the pressure to disclose. At the same time, disclosure must remain a safe and voluntary option for those who require more individualized support. By aligning inclusive language, policies, and everyday practices, organizations can create environments where neurodivergent employees feel valued, supported, and empowered to thrive—regardless of whether they choose to disclose.
